# *Gardenia jasminoides* fruit extract ameliorates non-alcoholic steatohepatitis with fibrosis by modulating inflammatory and fibrogenic pathways

**DOI:** 10.1371/journal.pone.0333800

**Published:** 2025-10-03

**Authors:** Natnicha Suttawong, Namthip Witayavanitkul, Maneerat Chayanupatkul, Natcha Wanpiyarat, Pornpen Werawatganone, Prasong Siriviriyakul, Duangporn Werawatganon

**Affiliations:** 1 Center of Excellence in Alternative and Complementary Medicine for Gastrointestinal and Liver Diseases, Department of Physiology, Faculty of Medicine, Chulalongkorn University, Bangkok, Thailand; 2 Department of Pathology, Faculty of Medicine, Chulalongkorn University, Bangkok, Thailand; 3 Department of Pharmaceutics and Industrial Pharmacy, Faculty of Pharmaceutical Sciences, Chulalongkorn University, Bangkok, Thailand; Guangdong Nephrotic Drug Engineering Technology Research Center, Institute of Consun Co. for Chinese Medicine in Kidney Diseases, CHINA

## Abstract

Despite advances in drug development, treatment for non-alcoholic steatohepatitis (NASH)-related liver fibrosis remains limited, underscoring the need for more effective therapeutic strategies. *Gardenia jasminoides* exhibits promising anti-inflammatory and anti-fibrotic properties, suggesting its potential as a therapeutic candidate for NASH-related liver fibrosis. Accordingly, this study aimed to investigate the therapeutic potential of *Gardenia jasminoides* fruit extract (GJE) in a rat model of NASH with fibrosis induced by a high-fat, high-fructose (HFHF) diet, and to elucidate the underlying molecular mechanisms. Male Sprague-Dawley rats were randomly assigned to four groups (n = 6/group): 1) Control group, fed a standard diet; 2) NASH with fibrosis group (NF), fed a HFHF diet for 20 weeks; 3) Low-dose GJE group (NF + Low dose GJE), treated with 0.119 g/kg BW of GJE; and 4) High-dose GJE group (NF+High dose GJE), treated with 0.239 g/kg BW of GJE. GJE was administered via intragastric gavage once daily from weeks 13–20. Serum markers of liver injury (ALT and AST) were assessed. Liver histology was examined to evaluate NASH and fibrosis severity. Immunohistochemistry was performed to detect NF-kB p65 and Col1a1 expression and protein levels of MMP-12, α-SMA, IL-13 and TGF-β1 were quantified. Serum ALT and AST levels were significantly increased in the NF group, which also showed the highest NASH histopathology scores and collagen deposition. GJE treatment significantly reduced ALT and AST levels in both low- and high- dose groups. The low-dose GJE group showed marked improvement in NASH histopathology, while both treatment groups exhibited reduced hepatic collagen deposition. GJE treatment significantly suppressed NF-kB p65 and Col1a1 expression, along with downregulation of MMP-12, α-SMA, IL-13 and TGF-β1 protein levels. In conclusion, GJE effectively ameliorated HFHF diet-induced NASH with fibrosis by modulating inflammatory and fibrogenic pathways.

## Introduction

Non-alcoholic fatty liver disease (NAFLD), also known as metabolic dysfunction-associated steatosis liver disease, is a metabolic disorder driven by factors such as obesity, diabetes, insulin resistance, hyperlipidemia, and high-fat, high-sugar diets [1]. It affects an estimated 25% of the global population [2]. Steatosis can progress to non-alcoholic steatohepatitis (NASH), a condition driven by sustained hepatic fat accumulation and inflammation. If left untreated, NASH may advance to liver fibrosis, cirrhosis, and ultimately hepatocellular carcinoma (HCC) [3]. The rising global consumption of high-fat, high-sugar diets is a major contributor to the increasing prevalence of NASH and liver fibrosis. Fructose, widely available both naturally and as a common additive in processed foods, plays a particularly harmful role [4]. Excessive fructose intake has been shown to promote greater hepatic fat accumulation and inflammation compared to other sugars, thereby accelerating the progression of NASH-related liver fibrosis [5].

*Gardenia jasminoides,* a traditional herbal medicine widely used in East Asia and included in the Chinese herbal pharmacopeia, conforms to the theory of food & medicine homology, possessing pharmacological and nutritional compounds that can aid in the treatment of liver fibrosis in China [6,7]. *In vivo* study using bile duct-ligated rats and *in vitro* study on human hepatic stellate cells (HSCs) have shown that *Gardenia jasminoides* treatment (50 and 100 mg/kg) significantly reduced collagen accumulation and suppressed the expression of transforming growth factor beta 1 (TGF-β1), collagen 1 (Col I) and alpha smooth muscle actin (α-SMA) [7]. In Thailand, the fruit of *Gardenia jasminoides*, known as a major source of natural yellow pigment due to its high carotenoid content [8], is commonly used in foods, beverages, and as a natural additive. Recent findings from our laboratory further support the therapeutic potential of *Gardenia jasminoides* fruit extract (GJE), particularly in animal models of gastrointestinal disorders, including gastropathy [9], hepatotoxicity [10], and gastritis [11]. GJE was selected in this study due to its established hepatoprotective properties, largely attributed to geniposide, a major iridoid glycoside component [12]. Geniposide has been widely studied for its bioactivity [12] and identified as a key contributor to GJE’s therapeutic effects. It has shown efficacy in various liver conditions, including cholestatic liver injury [13], NAFLD [14], NASH [15], liver fibrosis [16], and even liver cancer [17]. In our previous study, GJE containing 400 mg/kg of geniposide demonstrated protective effects against non-steroidal anti-inflammatory drugs (NSAID)-induced gastropathy in rats by downregulating inducible nitric oxide synthase (iNOS) and transcription factor nuclear factor kappa-B (NF-κB) under acute conditions [9]. Additionally, GJE treatment reduced acetaminophen (APAP)-induced hepatotoxicity by lowering serum alanine aminotransferase (ALT) and aspartate aminotransferase (AST) levels, increasing hepatic glutathione (GSH) levels (at 100–200 mg/kg geniposide), and reducing tumor necrosis factor-alpha (TNF-α) and malondialdehyde (MDA) levels (at 200 mg/kg geniposide) in mice [10]. GJE has also been shown to alleviate *Helicobacter pylori*-induced gastritis through inhibition of bacterial growth, reduction of inflammation, and enhancement of mucosal defenses following daily treatment for one week with GJE containing geniposide at doses of 8, 16, and 32 mg [11]. Several studies have reported the dose- and time dependent effects of geniposide under various liver injury models. In an acute model, geniposide (20 mg/kg) administered 30 minutes prior to ischemia/reperfusion (I/R) injury exhibited hepatoprotective effects via anti-inflammatory, anti-apoptotic, and antioxidant mechanisms in rats [18]. In bile duct ligation (BDL) model, geniposide (50 mg/kg) administered for 7 days before and after surgery significantly improved liver fibrosis in mice by reducing collagen deposition and suppressing α-SMA expression, as confirmed by histological staining and western blot analysis [13]. In a chronic model, long-term geniposide treatment (100 and 150 mg/kg) for six weeks in carbon tetrachloride (CCl_4_)-induced liver fibrosis in mice also downregulated α-SMA expression [19].

However, the anti-fibrotic potential of GJE in NASH-related liver fibrosis and its underlying mechanisms remain unexplored. This study aimed to investigate the therapeutic effects of GJE in a HFHF diet-induced rat model of NASH with fibrosis, focusing on its modulation of inflammatory and fibrogenic pathways. Two doses of GJE (0.119 and 0.239 g/kg body weight), corresponding to geniposide contents of 50 and 100 mg/kg, respectively were selected based on prior studies demonstrating the therapeutic efficacy of GJE and its principal active compound, geniposide.

## Materials and methods

### Experimental design

Twenty-four 6-week-old male Sprague-Dawley (SD) rats weighing from 180-200g were purchased from Nomura Siam International Co., Ltd., Bangkok, Thailand. The study protocol was approved by the Institutional Animal Care and Use Committee (IACUC), Faculty of Medicine, Chulalongkorn University (Animal ethics approval no.2491004). These rats were accommodated in a controlled environment with a 12-hour light and dark cycle at a temperature of 25 ± 1°C. Following a week-long acclimatization period, rats were divided into four groups (n = 6/group).

(1) Control group, fed a standard diet for 20 weeks
(2) NASH with fibrosis group (NF), fed a high-fat, high-fructose (HFHF) diet (55% fat, 35% carbohydrates [20% fructose, 15% starch], and 10% protein) for 20 weeks to induce NASH with fibrosis
(3) Low-dose GJE group (NF + Low dose GJE), fed the HFHF diet for 20 weeks and treated with low-dose GJE (0.119 g/kg body weight (BW), equivalent to 50 mg/kg BW geniposide) by intragastric administration once daily from weeks 13–20.
  (4) High-dose GJE group (NF+High dose GJE), fed the HFHF diet for 20 weeks and treated with high-dose GJE (0.239 g/kg BW, equivalent to 100 mg/kg BW geniposide) by intragastric administration once daily from weeks 13–20.

### Sample size calculation

The number of animals per group was determined using G*power software (version 3.1.9.7). The α-SMA expression data (mean ± SEM) reported by Lee J. A. et.al., [20] were used as reference values for the sample size estimation: Control, 0.91% ± 0.13; thioacetamide (TAA), 6.24% ± 0.39; Gardeniae Fructus (GF, 100 mg/kg), 4.81% ± 0.39; and silymarin (100 mg/kg), 3.77% ± 0.26. Based on these values, the sample size was calculated in G*Power, with α = 0.05 and power = 0.99., yielding six animals per group (total n = 24). The detailed G*Power calculation is provided in the Supporting information (S1 Fig).

### Animal welfare and method of sacrifice

Animals were provided ad libitum access to water and either a standard chow or a HFHF diet, according to their assigned experimental groups, for a total of 20 weeks. Body weight was recorded weekly, and weekly body weights for all groups are shown in the Supporting information (S2 Fig). From weeks 13–20, animals received GJE via oral gavage once daily for 8 weeks. Following each administration, animals were observed for 5–10 minutes for signs of labored breathing or distress [21–23] by trained research personnel. All researchers involved in animal handling had completed both theoretical and practical training certified by the Chulalongkorn University Laboratory Animal Center (CULAC). Animal health and welfare were monitored twice daily for clinical signs of pain, distress, or abnormal behavior. Humane endpoint criteria included signs of inflection, chronic inflammation, impaired mobility, abnormal resting posture, red periorbital staining, labored breathing, restlessness, unresponsiveness, failure to groom (resulting in an unkempt appearance), or a body weight loss exceeding 20% [24]. Animals exhibiting any signs of these criteria were promptly administered with analgesics (e.g., tramadol) to alleviate suffering and/or immediately euthanized via intraperitoneal injection of sodium thiopental (>50 mg/kg). No premature or unanticipated deaths occurred throughout the study.

At the end of the experiment, all animals were fasted overnight (8 hours) prior to euthanasia. An overdose of sodium thiopental (>50 mg/kg) was administered intraperitoneally for euthanasia, and the depth of anesthesia was confirmed by the absence of toe and tail pinch responses. Experimental procedures were initiated only after full anesthesia was confirmed. Blood collected via cardiac puncture was processed to isolate serum, which was stored at −80ºC until analysis of ALT and AST levels using a biochemical analyzer. Whole liver tissues were immediately excised and divided for histological and molecular analyses. For histological examination, portions of the liver were fixed in 10% neutral buffered formaldehyde and evaluated by a blinded, experienced pathologist. Paraffin-embedded liver sections were used for immunohistochemical detection of NF-kB p65 and collagen type 1 alpha 1 (Col1a1), while fibrosis staging was assessed using Sirius Red staining. The remaining liver tissues were snap-frozen in liquid nitrogen and stored at −80ºC for protein analysis. Expression levels of matrix metalloproteinases-12 (MMP-12), α-SMA, interleukin-13 (IL-13) and TGF-β1 were determined by Western blotting.

### Preparation of *Gardenia jasminoides* fruit extract

Dried *Gardenia jasminoides* fruit was purchased from Vejpong Pharmacy Co., Ltd., Bangkok, Thailand. The plant material was authenticated by Asso. Prof. Chaisak Chansriniyom, Ph.D, a qualified botanist, and the voucher specimen (CC-MS-0367) was deposited at the herbarium of the Department of Pharmacognosy and Pharmaceutical Botany, Faculty of Pharmaceutical Sciences, Chulalongkorn University, Thailand. Ten grams of the fruit were ground and soaked in 50 mL of water for 60 minutes. The soaked plant material was filtered, and the crude was soaked again in 50 mL of 50% ethanol for another 60 minutes and filtered. Filtrates from both extractions were combined and evaporated using a rotary evaporator (R-300 BUCHI Rotavapor®, Flawil, Switzerland). Geniposide present in the dried extract was quantified using high-performance liquid chromatography (HPLC) analysis which was modified from Tang et al. [25]. Geniposide standard was obtained from Sigma Aldrich, St. Louis, MO, USA. The HPLC system (Shimadzu LC20A®, Tokyo, Japan) was equipped with a C18 column (5 µm), 150 × 4 mm (Agilent®, Santa Clara, CA, USA), and an ultraviolet detector. The elution profile was 10% acetonitrile at 0 minute, the linear gradient to 18% acetonitrile ranged from 0 to 15 minutes, the linear gradient to 28% acetonitrile ranged from 15 to 20 minutes, the linear gradient to 38% acetonitrile ranged from 20 to 40 minutes, the linear gradient to 50% acetonitrile ranged from 40 to 50 minutes, the final elution at 50% acetonitrile ranged from 50 to 55 minutes, and the linear gradient to 10% acetonitrile ranged from 55 to 64 minutes. The flow rate was 1 mL/min, and the injection volume was 10 µL. Geniposide was detected at 238 nm. These results are consistent with previous reports identifying 238 nm as the characteristic absorption wavelength for geniposide [26]. After analysis, the dried extract was stored at −20°C for further study. The extract dosage was determined based on the target amount of geniposide. The dose of GJE was calculated based on its geniposide content. The calculation was performed using the following formula:


$$\text{GJE dose }(\text{mg})\;=\;\frac{\text{Body weight }(\text{kg})\;\times \text{ Desired geniposide dose }(\text{mg}/\text{kg})\;\times \text{ 1000}}{\text{Geniposide yield }(\text{mg}/\text{g of GJE})}$$

In this study, the geniposide yield of GJE was 12.53% (125.3 mg per 1000 mg of GJE). For a 300 g rat (0.3 kg body weight), the dose of 50 mg/kg of geniposide corresponded to 15 mg of geniposide, equivalent to 119 mg of GJE (0.119 g.). The high dose of 100 mg/kg of geniposide corresponded to 30 mg of geniposide, equivalent to 239 mg of GJE (0.239 g.)

Both low and high doses of the dried extract were dissolved in distilled water prior to administration to rats in both treatment groups. The rationale for selecting the geniposide dosage was influenced by Ma et al.‘s research, where they induced NASH using a high-fat diet and administered geniposide at doses of 25, 50, and 100 mg/kg body weight. They determined that doses of 50 and 100 mg/kg were effective in treating NASH [15]. A subchronic toxicity study estimated the LD50 of *Gardenia* extract to be over 0.5 g/kg/day. Rats given a dose of 0.5 g/kg/day showed no treatment-related changes during autopsy [25]. Thus, the GJE extract dosage used in this study falls within a safe range.

### Serum analysis

In this study, the biochemical analyzer Dri-Chem NX-600 (Fujifilm, Japan) was used to determine serum ALT and AST levels using a colorimetric principle. One drop of serum (10 µl) was pipetted onto a reagent strip before being inserted into the instrument. Then, serum ALT and AST levels were reported in units per liter (U/L).

### Liver histopathology

Liver tissues were fixed in 10% formalin for 24 hours, then processed using standard protocols and embedded in paraffin blocks. Sections were cut at a thickness of 3.5 μm and stained with hematoxylin and eosin (H&E) and Sirius Red staining. The severity of NASH was evaluated based on Brunt’s criteria [27,28], while fibrosis staging was assessed using the Metavir scoring system [29].

### Immunohistochemistry

Liver tissue sections underwent deparaffinization and rehydration. Following rinsing with running tap water, antigen retrieval was achieved by pre-treatment process of deparaffinization, rehydration and epitope retrieval (PT Link) treatment (Dako, Denmark) in citrate buffer at pH 9.0 for 20 minutes. To prevent false positive staining, 3% hydrogen peroxide (H_2_O_2_) was applied to block endogenous peroxidase activity for 5 minutes. Subsequently, slides were incubated with primary antibodies, specifically NF-kB p65 (diluted 1:400; Abcam, Cambridge, UK) and Col1a1 (diluted 1:100; Cell signaling, California, USA) for 1 hour at room temperature, followed by incubation with a secondary antibody for 30 minutes. Diaminobenzidine (DAB) (Dako, Denmark) served as the chromogenic substrate to develop a brown color indicative of specific antigens. Slides were counterstained with hematoxylin at appropriate time. Digital images for visualizing protein expression were obtained with a light microscope and analyzed using Aperio ImageScope software (Leica Biosystems Imaging, Inc., MD, USA) in 10 randomly selected fields at a 400X magnification. Positive stained cells were defined as brown color in the cytoplasm of hepatocytes. Furthermore, an automated pixel analysis was utilized to determine the ratio of strongly positive pixels to the total area detected [9]. The Col1a1 expression levels were quantified in each group using five randomly selected non-overlapping images. The images were imported into FIJI software (SciJava software ecosystem, Wisconsin, USA) for analysis. The positive region of each image was identified using automatic thresholding, followed by manual adjustment of lower and upper thresholds. This method ensured precise measurement of the area and integrated density of the positive regions, effectively focusing solely on the positive areas while excluding background or negative regions [30].

### Western blot analysis

Frozen liver tissue was thawed and homogenized on ice using tissue protein extraction reagent (T-PER; Thermo Fisher Scientific, USA) along with a proteinase and phosphatase inhibitor cocktail as provided in the instructions. Lysates were centrifuged at 16,000xg, 4ºC for 5 minutes. Then, the supernatants were gathered to determine the total protein concentration using a Bicinchoninic acid (BCA) protein assay kit (Pierce; Thermo Fisher Scientific, USA). Equal amounts of proteins were equally distributed and loaded onto a 10% sodium SDS-PAGE sorted according to their distinct molecular weights. Following the electrophoresis process, the proteins on the gel were then transferred to a polyvinylidene difluoride (PVDF) membrane (0.45 µm filter, BioRad, USA) using a wet tank method. The membranes were blocked with 1% bovine serum albumin (BSA, Merck, USA) for an hour at room temperature. Then, the membranes were incubated with primary antibodies targeting MMP-12 (1:1000, Novus Biologicals, Missouri, USA), α-SMA (1:1000, Cell Signaling, Massachusetts, USA), IL-13 (1:1000, Novus Biologiclas, Missouri, USA), TGF-β1 (1:1000, Abcam, Cambridge, USA), cyclophilin B (CPB; 1:20000, Abcam, Cambridge, USA), and beta actin (1:1000, Santa Cruz, California, USA) overnight at 4°C. After washing with phosphate-buffered saline solution with the detergent tween 20 (PBST), the membranes were incubated with HRP-conjugated anti-rabbit and mouse IgG secondary antibody for 90 minutes at room temperature. The protein signals were developed using clarity western enhanced chemiluminescence (ECL) blotting substrates (BioRad, California, USA). Subsequently, the Bio-Rad ChemiDoc Touch Imaging System was utilized to determine the protein bands of interest, and densitometry measurements were quantified using Image Lab software (BioRad, California, USA). Protein expression levels were normalized to CPB or beta actin levels as appropriate.

### Statistical analysis

Statistical analysis was conducted using SPSS software version 22.0 for Windows. Data were reported as mean ± standard deviation (SD). Differences in means across experimental groups were assessed using one-way analysis of variance (One-way ANOVA) followed by the LSD post-hoc test for continuous variables, and Chi-square tests were used to compare differences between groups for categorical variables. Descriptive statistics were employed for histological examinations. Statistical significance was defined as *p *< 0.05.

## Results

### Effects of GJE extract on liver morphology and histopathological changes

**Fig 1:**
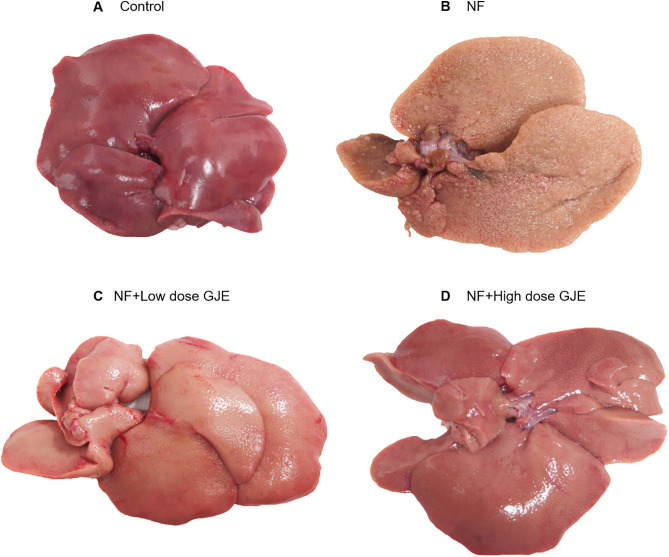
Effects of GJE extract treatment on liver appearance in each group. Control (A), NF (B), NF + Low dose GJE (C), and NF+High dose GJE (D) groups. Grossly, livers in the NF group appeared pale and rough, whereas GJE treatment (low and high doses) improved surface smoothness and restored a pink color compared to controls.

Gross liver appearance showed that the liver in the NF group was pale with rough surface when compared to dark red color with smooth surface in the control group. When treated with both low and high doses of GJE, a smoother liver surface and pink color was observed when compared to the NF group (Fig 1).

**Fig 2:**
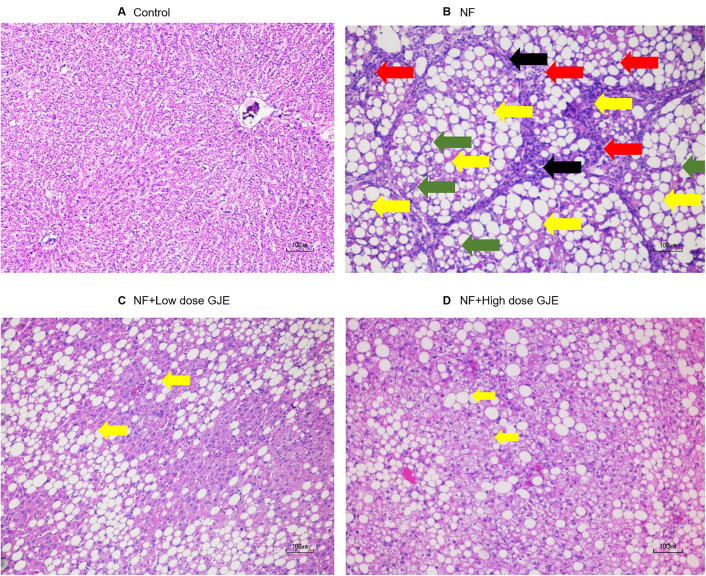
Effects of GJE extract treatment on NASH histopathology in each group. Control (A), NF (B), NF + Low dose GJE (C), and NF+High dose GJE (D) groups. H&E-stained sections, original magnification 10X. Yellow arrows indicated fat deposition in hepatocytes (steatosis); Red arrows indicated inflammatory cell infiltration in hepatic lobule (lobular inflammation); Green arrows indicated hepatocyte ballooning; Black arrows indicated bridging band of fibrosis between two portal tracts. H&E staining showed that NF livers had marked steatosis, lobular inflammation, and ballooning, while GJE treatment improved these features.

As shown in Fig 2, NASH features were assessed by H&E staining. Liver tissues from the NF group showed pronounced lipid droplets (yellow arrows), lobular inflammation (red arrows), and varying degrees of hepatocellular ballooning (green arrows) and bridging of fibrosis (black arrows) compared to the control group. In contrast, rats treated with low and high doses of GJE showed reduced steatosis, lobular inflammation and hepatocyte ballooning (Table 1). Liver fibrosis was evaluated using the Metavir scoring system (Table 2), with advanced fibrosis (F3/F4) observed in the NF group. GJE treatment at both doses significantly attenuated fibrosis severity compared to the NF group.

**Table 1:** The liver histopathology scores for all experimental groups following Brunt’s criteria.

Group	Number of rats (N)	The liver histopathology scores following Brunt’s criteria										
		Steatosis				Ballooning			Lobular inflammation			
		0	1	2	3	0	1	2	0	1	2	3
Control	N = 6	6	–	–	–	6	–	–	6	–	–	–
NF	N = 6	–	–	–	6	2	2	2	–	4	1	1
NF + Low dose GJE	N = 6	1	1	1	3	2	3	1	1	1	3	1
NF+High dose GJE	N = 6	–	–	2	4	–	6	–	–	1	3	2

Data are expressed as the number of rats in each liver’s histopathology grading score according to Brunt’s criteria. Steatosis was scored from 0 to 3 as follows; 0 = absence of fat accumulation in hepatocyte, 1 = 5% − 33% of fat accumulation in hepatocyte, 2 = > 33% − 66% of fat accumulation in hepatocyte, and 3 = > 66% of fat accumulation in hepatocyte. Ballooning was scored from 0 to 2; 0 = absence of ballooning in hepatocyte, 1 = few ballooning cells and 2 = many cells or prominent ballooning. Lobular inflammation was scored from 0 to 3; 0 = absence of inflammation, 1 = < 2 foci per 200x field, 2 = 2–4 foci per 200x field and 3 = > 4 foci per 200x field.

**Table 2:** The classification of liver fibrosis stage for all experimental groups assessed by Metavir scoring system.

Group	Number of rats (N)	The liver fibrosis stage following Metavir scoring systems				
		F0	F1	F2	F3	F4
Control	N = 6	6	–	–	–	–
NF	N = 6	–	–	–	3	3
NF + Low dose GJE	N = 6	4	1	1	–	–
NF+High dose GJE	N = 6	2	1	–	3	–

Data are expressed as the number of rats in each fibrosis grade for all experimental groups. Liver fibrosis was assessed using the Metavir scoring system as follows; Fibrosis score: F0 = No fibrosis can be detected, F1 = Fibrosis exists with expansion of portal zones, F2 = Fibrosis exists with expansion of most portal zones, and occasional bridging, F3 = Fibrosis exists with expansion of most portal zones, marked bridging, and occasional modules and F4 = Presence of cirrhosis, *p *< 0.05 for overall comparison and Pearson Chi-square tests were used to compare differences between groups for categorical variables.

**Fig 3:**
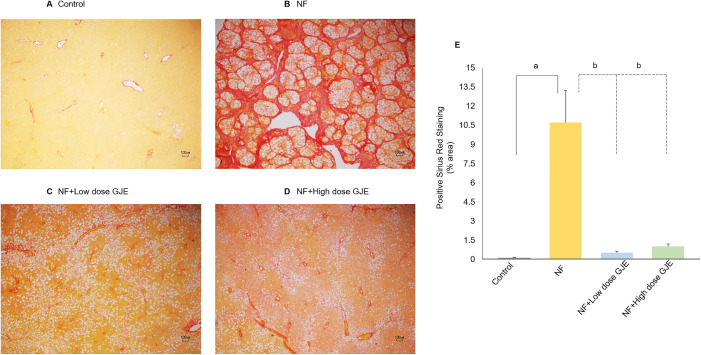
Effects of GJE extract treatment on histopathology of liver fibrosis in each group. Control (A), NF (B), NF + Low dose GJE (C), and NF+High dose GJE (D) groups. Sirius Red stained sections, original magnification 4X. Sirius Red staining showed a marked increase in collagen deposition in the NF group versus controls, which was significantly reduced by both low and high doses of GJE. The percent positive staining area increased in the NF group versus controls (10.71 ± 6.18% vs. 0.12 ± 0.06%, p < 0.05) and was reduced by low and high GJE doses (0.52 ± 0.31% and 1.00 ± 0.43%, respectively, *p* < 0.05). The positive Sirius Red staining (%area) (E). Data are expressed as mean ± SD, error bars indicate standard deviation, n = 6/group. a: when compared to the control group. b: when compared to the NF group, *p* < 0.05 for both a and b.

Liver fibrosis is characterized by the accumulation of type I collagen in the liver. This parameter was evaluated using the Sirius Red staining method. The percentage of Sirius Red-positive staining relative to the total area was calculated for all experimental groups to assess the extent of liver fibrosis. The percent positive staining area significantly increased in the NF group compared to the control group (10.71 ± 6.18% vs. 0.12 ± 0.06%, *p* < 0.05). Treatment with GJE at both low and high doses significantly reduced the percent positive staining area when compared to the NF group (0.52 ± 0.31% vs. 1.00 ± 0.43% vs. 10.71 ± 6.18%, respectively, *p* < 0.05) (Fig 3).

### Effects of GJE extract on serum transaminases

Serum ALT and AST levels in the NF group significantly increased compared to the control group (ALT; 27.40 ± 7.86 U/L vs. 18.60 ± 1.67 U/L, and AST; 110.50 ± 22.87 U/L vs. 80.00 ± 18.57 U/L, respectively, *p *< 0.05)

**Fig 4:**
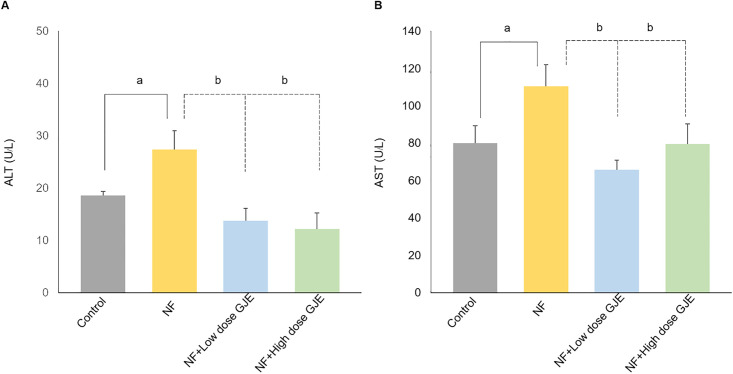
Effects of GJE extract treatment decreased serum ALT and AST levels. Serum ALT (A) and AST (B) levels in all experiment groups. Serum ALT and AST levels were elevated in the NF group compared to controls and were significantly reduced by both low and high GJE doses. Data are expressed as mean ± SD, error bars indicate standard deviation, n = 6/group. a: when compared to the control group. b: when compared to the NF group, **p* *< 0.05 for both a and b.

After treated with low and high doses of GJE, serum ALT and AST levels significantly decreased when compared to the NF group (ALT; 13.80 ± 5.26 U/L vs. 12.20 ± 6.90 U/L vs. 27.40 ± 7.86 U/L, and AST; 65.75 ± 10.50 U/L vs. 79.50 ± 21.55 U/L vs. 110.50 ± 22.87 U/L, respectively, *p *< 0.05), as shown in Fig 4.

### Effects of GJE extract on inflammatory markers

**Fig 5:**
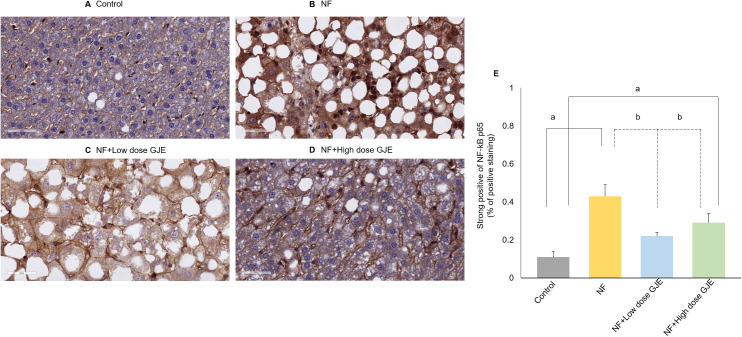
Effects of GJE extract treatment decreased expression of NF-kB p65. Control (A), NF (B), NF + Low dose GJE (C), and NF+High dose GJE (D) groups using immunohistochemistry (40X magnification). NF-κB p65 staining was higher in the NF group than controls (0.43 ± 0.11% vs. 0.11 ± 0.05%, **p* *< 0.05) and decreased with low and high GJE doses (0.22 ± 0.04% and 0.29 ± 0.10%, respectively, **p* *< 0.05), though the high-dose group remained elevated versus controls (0.29 ± 0.10% vs. 0.11 ± 0.05%, **p* *< 0.05). Brown color represents NF-kB p65 positive staining. The percentage of positive staining for NF-kB p65 (E). Data are expressed as mean ± SD, error bars indicate standard deviation, n = 6/group. a: when compared to the control group. b: when compared to the NF group, *p* < 0.05 for both a and b.

The percentage of positive staining for NF-kB p65 in the NF group was significantly higher when compared to the control group (0.43 ± 0.11% vs. 0.11 ± 0.05%, *p *< 0.05). A significantly decreased expression was detected in both low and high groups compared to the NF group (0.22 ± 0.04% vs. 0.29 ± 0.10% vs. 0.43 ± 0.11%, respectively, *p *< 0.05). However, NF-kB p65 expression in the NF+High dose GJE group remained significantly higher than in the control group (0.29 ± 0.10% vs. 0.11 ± 0.05%, *p* < 0.05) (Fig 5).

### Effects of GJE extract on NASH-related liver fibrosis markers

**Fig 6:**
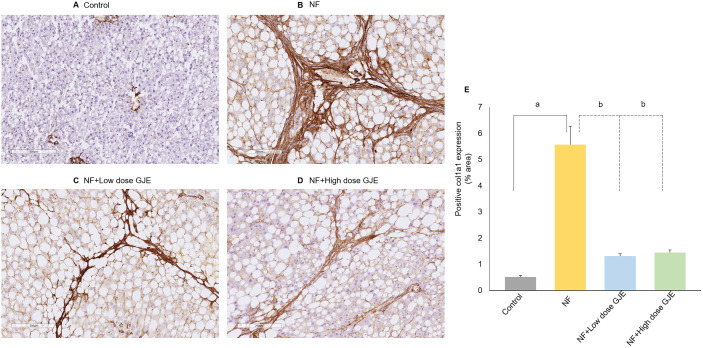
Effects of GJE extract treatment decreased accumulation of Col1a1. Control (A), NF (B), NF + Low dose GJE (C), and NF+High dose GJE (D) groups using immunohistochemistry (10X magnification). Col1a1-positive areas were significantly increased in the NF group compared to controls and were markedly reduced by both low and high GJE doses. The positive areas of Col1a1 expression (%area) (E) in all experimental groups. Data are expressed as mean ± SD, error bars indicate standard deviation, n = 6/group. a: when compared to the control group. b: when compared to the NF group, **p* *< 0.05 for both a and b.

The percentage of positive areas of Col1a1 in the NF group significantly increased when compared to the control group (5.57 ± 1.72% vs. 0.51 ± 0.15%, respectively; *p *< 0.05), A significantly decreased expression was observed in both NF + Low and NF+High doses of GJE groups when compared to the NF group (1.31 ± 0.28% vs. 1.44 ± 0.26% vs. 5.57 ± 1.72%, respectively; *p* < 0.05) (Fig 6).

**Fig 7:**
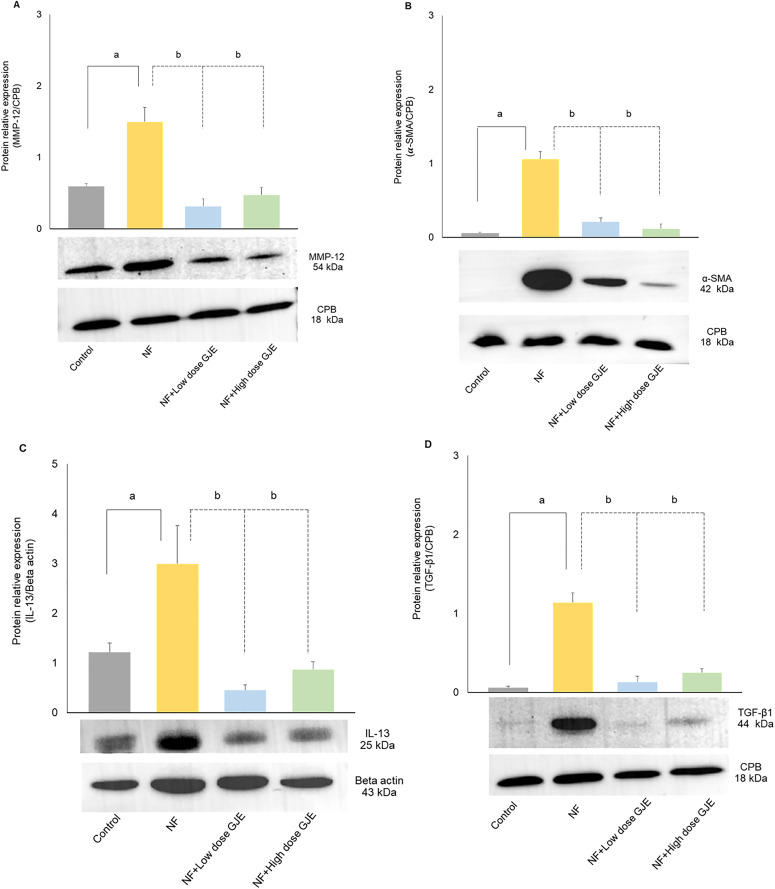
Effects of GJE extract treatment decreased protein expression of MMP-12, α-SMA, IL-13, and TGF-β1 levels. Protein expressions of MMP-12 (A), α-SMA (B), IL-13 (C), and TGF-β1 (D). Hepatic MMP-12, α-SMA, IL-13 and TGF-β1 expressions were elevated in the NF group compared to controls and were significantly reduced by both low and high GJE doses. Data are expressed as mean ± SD, error bars indicate standard deviation, n = 6/group. a: when compared to the control group. b: when compared to the NF group, **p* *< 0.05 for both a and b.

The hepatic protein expressions of MMP-12, α-SMA, IL-13, and TGF-β1 significantly increased in the NF group when compared to the control group (MMP-12; 1.50 ± 0.49 vs. 0.60 ± 0.07, α-SMA; 1.06 ± 0.26 vs. 0.06 ± 0.03, IL-13; 3.00 ± 1.86 vs. 1.22 ± 0.44, and TGF-β1; 1.14 ± 0.27 vs. 0.06 ± 0.04, respectively, *p* < 0.05). Treatment with GJE at both low and high doses significantly decreased MMP-12, α-SMA, IL-13 and TGF-β1 protein expressions compared to the NF group (MMP-12; 0.32 ± 0.15 vs. 0.48 ± 0.15 vs. 1.50 ± 0.49, α-SMA; 0.21 ± 0.12 vs. 0.12 ± 0.13 vs. 1.06 ± 0.26, IL-13; 0.46 ± 0.25 vs. 0.88 ± 0.37 vs. 3.00 ± 1.86, and TGF-β1; 0.14 ± 0.15 vs. 0.25 ± 0.10 vs. 1.14 ± 0.27, respectively, *p* < 0.05) (Figs 7A–D). Western blot analysis results are included in Supporting information (S3 File).

## Discussion

This study was the first to evaluate the therapeutic effects of GJE extract in a HFHF diet-induced model of NASH with fibrosis. Our findings indicated that the extract alleviated HFHF-induced NASH and fibrosis by reducing liver inflammation, and fibrosis severity.

NASH occurs due to the excessive accumulation of triglycerides in hepatocytes, triggering lipotoxicity and oxidative stress, which lead to liver inflammation and eventually progress into NASH. In this study, a HFHF diet was chosen to induce NASH with fibrosis, as it more closely resembles current human dietary patterns. Fat and fructose can cause cellular damage, leading to liver inflammation [31]. In animals, high fructose consumption was linked to an upregulation of lipogenic genes, including acetyl-CoA carboxylase 1 (Acc1), fatty acid synthase (FAS), and stearoyl CoA desaturase (SCD1), compared to a high fat diet alone. Additionally, fructose metabolites can directly activate transcription factors sterol regulatory element binding protein (SREBP)-1c and carbohydrate-response element binding protein (ChREBP), thereby promoting hepatic lipogenesis [32]. Fructokinase is a crucial enzyme that converts fructose into fructose-1-phosphate and produces acetyl-CoA, a precursor essential for triglyceride synthesis in the liver [33]. Consistent with previous findings [34,35], our study demonstrated that rats fed with a HFHF diet for 20 weeks developed characteristic histological features of NASH, including marked steatosis, hepatocellular ballooning, lobular inflammation, and fibrosis.

Our results showed that serum ALT and AST levels, indicators of liver injury, significantly increased in the NF group when compared to the control group. A previous study similarly showed the increased ALT and AST levels in the NASH group fed a high fat diet compared to the control group [15]. A prior study demonstrated that lipopolysaccharide (LPS) activates toll-like receptor 4 (TLR4), thereby activating Kupffer cells. High fructose intake enhances the synthesis of saturated fatty acids, further stimulating TLR4 receptors in the liver [36]. Once activated, these receptors trigger the NF-κB pathway, leading to oxidative stress in hepatocytes by promoting the release of pro-inflammatory cytokines, such as TNF-α, from Kupffer cells, which may lead to liver tissue damage and facilitate the progression of NASH [15].

α-SMA is a specific actin that serves as a dependable indicator for activated HSCs and myofibroblasts which produce fibrotic tissue [37]. Data by Sun et al. demonstrated that increased α-SMA and TGF-β1 expression was observed in thioacetamide (TAA) induced liver fibrosis in a rat model [38]. Lu Yang et al., demonstrated that CCl_4_ administration can induce liver fibrosis by increasing the expression of α-SMA [16]. In the present study, we found significantly higher fibrosis stages, as determined by the Metavir scoring system, and increased expression of Col1a1 in the NF group compared to the control group.

Geniposide has previously been shown to have significant effects on reducing inflammation, apoptosis, oxidative stress and fibrosis [39]. Our study demonstrated that GJE extract, with geniposide as its primary constituent, exerted a more pronounced anti-inflammatory effect than an anti-steatotic one, as evidenced by a significant reduction in serum ALT and AST levels in both treatment groups compared to the NF group, despite no observable differences in the degree of steatosis. Moreover, after treatment with both low and high doses of GJE, NF-kB p65 expression significantly decreased when compared with the NF group. A previous study on NSAID-induced gastropathy in rats found that GJE extract could reduce the expression of iNOS and NF-κB [9]. However, this study shows that the high-dose GJE treated group still had significantly higher expression of NF-kB p65 when compared to the control group. This finding suggested that the higher dose was not superior to the lower dose in its anti-inflammatory effects.

Our study demonstrated that GJE treatment in both low and high doses could reduce hepatic collagen deposition and decrease the expression of Col1a1. Tingting Qin, et al., showed that geniposide could reduce Col1a1 expression in a BDL induced fibrosis model [13]. They found that geniposide could normalize bile acid levels and suppress NOD-like receptor protein 3 (NLRP3) inflammasome activation. This occurred through the activation of sirtuin 1 (SIRT1) indirectly, leading to the reversal of farnesoid X receptor (FXR) acetylation. These mechanisms played a role in the protective effects of geniposide against BDL-induced hepatic fibrosis in mice [13]. We also found that both low and high doses of GJE extract could decrease the expression of MMP-12 levels which might explain the anti-fibrotic effect of GJE. MMP-12 is an enzyme released by macrophages that plays a role in suppressing the activity of other MMP subtypes, including MMP-2 and MMP-13, which contribute to the degradation of extracellular matrix (ECM) deposits and the reduction of IL-13 release [40].

Treatment with the GJE, both low and high doses, significantly reduced the upregulation of α-SMA protein expression when compared to the NF group. Treatment with geniposide has been shown to decrease α-SMA expression, thereby mitigating liver fibrosis [16]. Interestingly, this study shows that the high dose group showed a greater reduction in α-SMA expression compared to the low dose group, albeit not statistically significant. IL-13 directly stimulates the production of collagen I and other essential genes associated with fibrosis, such as α-SMA and connective tissue growth factor (CTGF) in HSCs. These cells are the primary source of ECM during the development of liver fibrosis [41]. Previous study showed that IL-13 protein expression was upregulated in a rat model of bilateral ovariectomized plus HFHF fed induced NASH with fibrosis, suggesting that IL-13 plays a significant role in fibrogenesis [34]. This study demonstrated that GJE treatment could reduce the expression of IL-13. Therefore, GJE might exert its anti-fibrogenic effect through the reduction of IL-13 protein expression.

Elevated TGF-β1 levels closely correlate with the severity of NAFLD and liver fibrosis [42,43]. TGF-β1 is strongly associated with liver inflammation, promoting collagen-1 production and driving the activation of myofibroblasts [44]. High serum TGF-β1 levels have therefore been proposed as a potential biomarker of fibrosis [45]. Moreover, adipocytes can also release multiple pro-inflammatory cytokines, including TGF-β, IL-6, and TNF-α [46]. Additionally, liver diseases are associated with the accumulation of hepatic natural killer (NK) cells, which produce IL-4 and IL-13. Then, IL-3 further stimulates HSCs to release and regulate TGF-β expression [47]. Therefore, TGF-β1 plays a significant factor in accelerating disease progression, which in turn enhances the production of ECM [48]. This consequently leads to the development of liver fibrosis [49]. In the present study, we found that administration of GJE at both low and high doses markedly suppressed the increased expression of TGF-β1 protein relative to the NF group.

Although both low and high doses of GJE were tested, no clear dose-dependent effect was observed across the measured parameters, as differences between doses were not statistically significant. Low-dose GJE appeared slightly more effective in improving certain histopathological features (e.g., ballooning and lobular inflammation), whereas high-dose GJE showed a modest trend toward reducing α-SMA. These variations suggest that the effective range of GJE (or its active component geniposide) may fall within both tested doses, and that higher doses do not necessarily confer additional benefit, possibly due to saturation of the underlying mechanisms or a slight metabolic burden.

However, our study has some limitations. Because we used whole fruit extract rather than purified geniposide, other constituents in the extract may have contributed to the observed effects, although HPLC analysis indicated that they were present only in small amounts. While geniposide was confirmed as the major compound in GJE, minor components such as iridoids and flavonoids could also play a role. For example, asperulosidic has been reported to exert anti-fibrotic effects in renal fibrosis [50]. Taken together, these findings suggest that geniposide is most likely the principal active compound, although contributions from other minor components cannot be completely excluded. To address this limitation, future studies should evaluate the specific effects of purified geniposide to confirm its role in mediating the observed hepatoprotective effects. Furthermore, food intake could not be measured individually because the animals were housed in groups. At the group level, consumption of the HFHF diet did not differ between NASH with fibrosis model groups with or without GJE treatment. After GJE administration, food intake remained unchanged, with no significant differences between treated and untreated NASH groups. Food intake in the control group was not assessed, as these rats were fed ad libitum. Importantly, weekly body weights were consistent with these findings (Supporting information, S2 Fig), indicating that the beneficial effects of GJE on NASH with fibrosis were not attributable to alterations in appetite or overall metabolic condition. However, an important strength of this study was the use of a HFHF diet alone to induce NASH with fibrosis, a model that has not been previously explored. This is a strongly defined model of liver fibrosis that closely resembles the human condition. Moreover, this research used GJE, a natural compound, to explore its potential as a new alternative treatment for NASH with fibrosis, a disease with limited therapeutic options. Our findings suggested that the effective dose for treating NASH with fibrosis was 0.119 g/kg BW of GJE extract in this animal model.

## Conclusion

**Fig 8:**
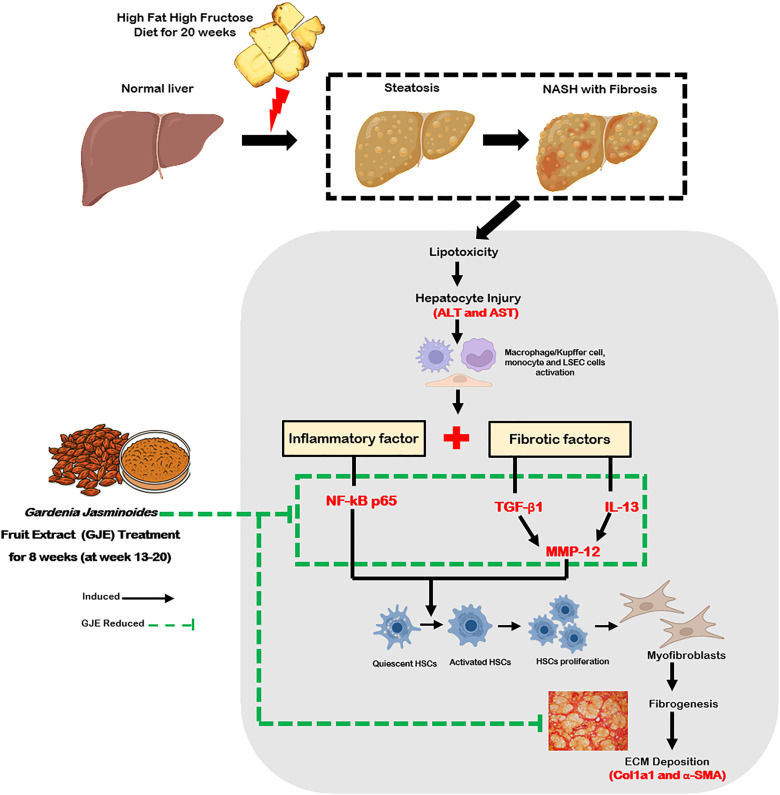
Proposed molecular mechanisms underlying GJE’s protective effects against NASH with fibrosis through modulation of inflammatory and fibrogenic signaling pathways. HFHF diets promotes hepatic fat accumulation, which can progress to inflammation and ultimately liver fibrosis. Excess hepatic lipids induce lipotoxicity, leading to hepatocyte injury. This damage activates macrophages, Kupffer cells, monocytes, and liver sinusoidal endothelial cells (LSECs), which in turn trigger inflammatory mediators such as NF-κB p65 and fibrogenic factors including TGF-β1, IL-13, and MMP-12. These signals stimulate hepatic stellate cells (HSCs) to proliferate and differentiate into myofibroblasts, resulting in fibrogenesis and extracellular matrix (ECM) deposition, as indicated by Col1a1 and α-SMA. Collectively, these pathways drive liver fibrosis. GJE may counteract this process by suppressing HFHF diet–induced inflammatory mediators and fibrogenic factors, thereby mitigating the progression of NASH with fibrosis.

In conclusion, the HFHF diet induced hepatic fat accumulation, lipotoxicity, and hepatocyte injury, which active immune cells and initiate inflammatory (NF-κB p65) and fibrotic (MMP-12, IL-13, TGF-β1) pathways. These cascades promoted HSC activation, myofibroblast differentiation, and ECM deposition (Col1a1, α-SMA), ultimately leading to liver fibrosis. Our findings suggest that GJE treatment ameliorated HFHF induced NASH with fibrosis primarily through anti-inflammatory and anti-fibrotic mechanisms, whereas its anti-lipogenic effects appeared less prominent (Fig 8). Further studies are warranted to confirm the therapeutic potential of GJE in clinical practice.

## Supporting information

S1 Fig Sample size calculation using G*Power software (version 3.1.9.7). As α-SMA expression was one of our primary outcomes, data from Lee J. A. et.al., [20] were used as the reference, with α = 0.05 and power = 0.99., yielding six animals per group (total n = 24).
(TIF)

S2 Fig Weekly body weights for all experimental groups. Control rats were fed a standard diet, whereas the NASH with fibrosis (NF) model was induced by a high-fat high-fructose (HFHF) diet. The graph shows weekly body weights for all groups over the 20-week period. Rats fed the HFHF diet exhibited lower body weights than controls from week 2 onward, with this difference was maintained throughout the study. However, no significant differences were observed among NF groups with or without GJE treatment. Data are expressed as mean ± SD (n = 6/group). a: *p* < 0.05 compared with the control group.
(TIF)

S3 File Original uncropped blot images showing detection bands for MMP-12, α-SMA, IL-13, TGF-β1, beta actin, and CPB. Lane 1 contains the PM2600 ExcelBand™ 3-Color High Range protein marker (SMOBIO, Taiwan), and lanes 2–5 correspond to Control, NF, NF + Low dose GJE, and NF+High dose GJE groups, respectively. Blots were developed using Clarity Western ECL substrates (BioRad, California, USA), captured with the Bio-Rad ChemiDoc Touch Imaging System, and quantified by Image Lab software (Bio-Rad, California, USA). Processed images were exported as TIF files for publication.
(PDF)

## Abbreviations

α-SMA: alpha-smooth muscle actin
Col1a1: collagen type 1 alpha 1
CPB: Cyclophilin B
IL-13: interleukin-13
MMP-12: matrix metalloproteinases-12
NASH: non-alcoholic steatohepatitis
NF-kB: nuclear factor-kB
LSEC cell: Liver Sinusoidal Endothelial Cell.
