# Supplementary figures and images for: Gardenia jasminoides fruit extract ameliorates non-alcoholic steatohepatitis with fibrosis by modulating inflammatory and fibrogenic pathways
Source: PLoS One. 2025 Oct 3;20(10):e0333800. doi: 10.1371/journal.pone.0333800 (PMC12494252; doi:10.1371/journal.pone.0333800)

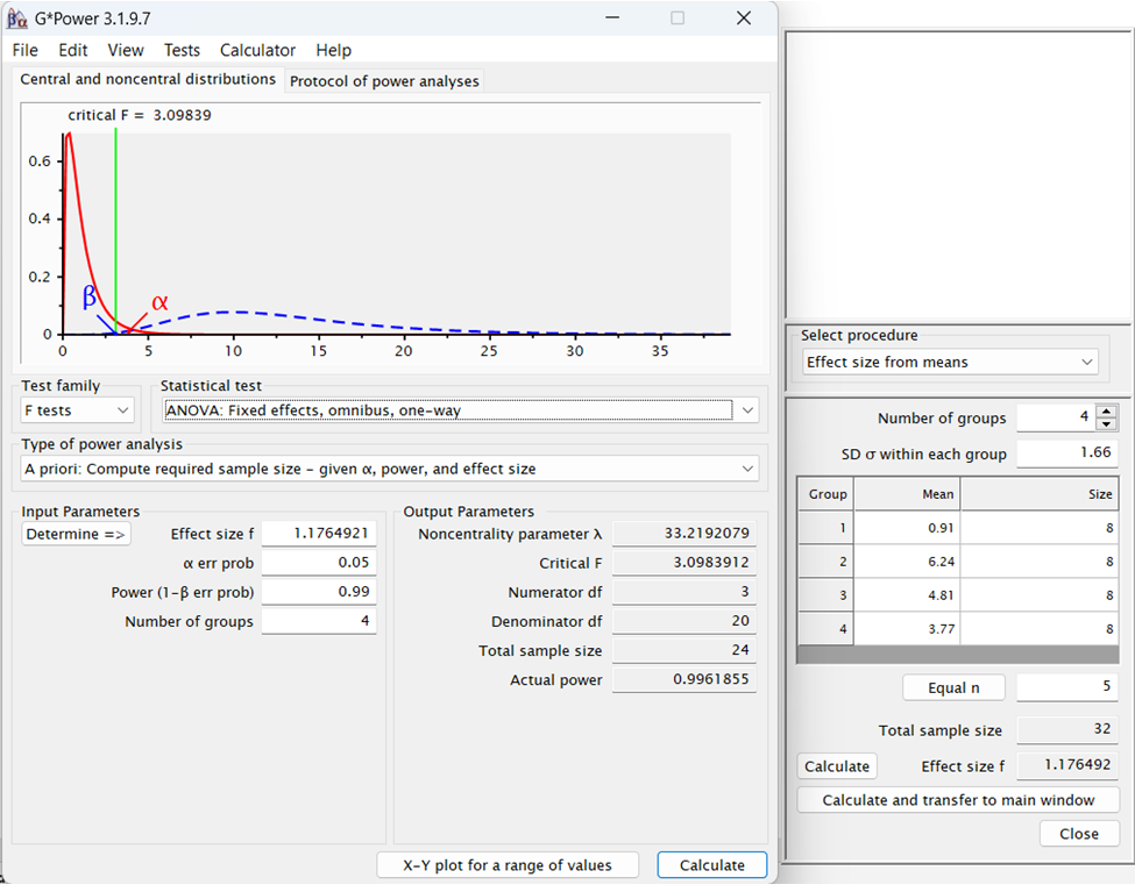

Supplement: S1 Fig — As α-SMA expression was one of our primary outcomes, data from Lee J. A. et.al., [20] were used as the reference, with α = 0.05 and power = 0.99., yielding six animals per group (total n = 24). (TIF) [file pone.0333800.s001.tif]

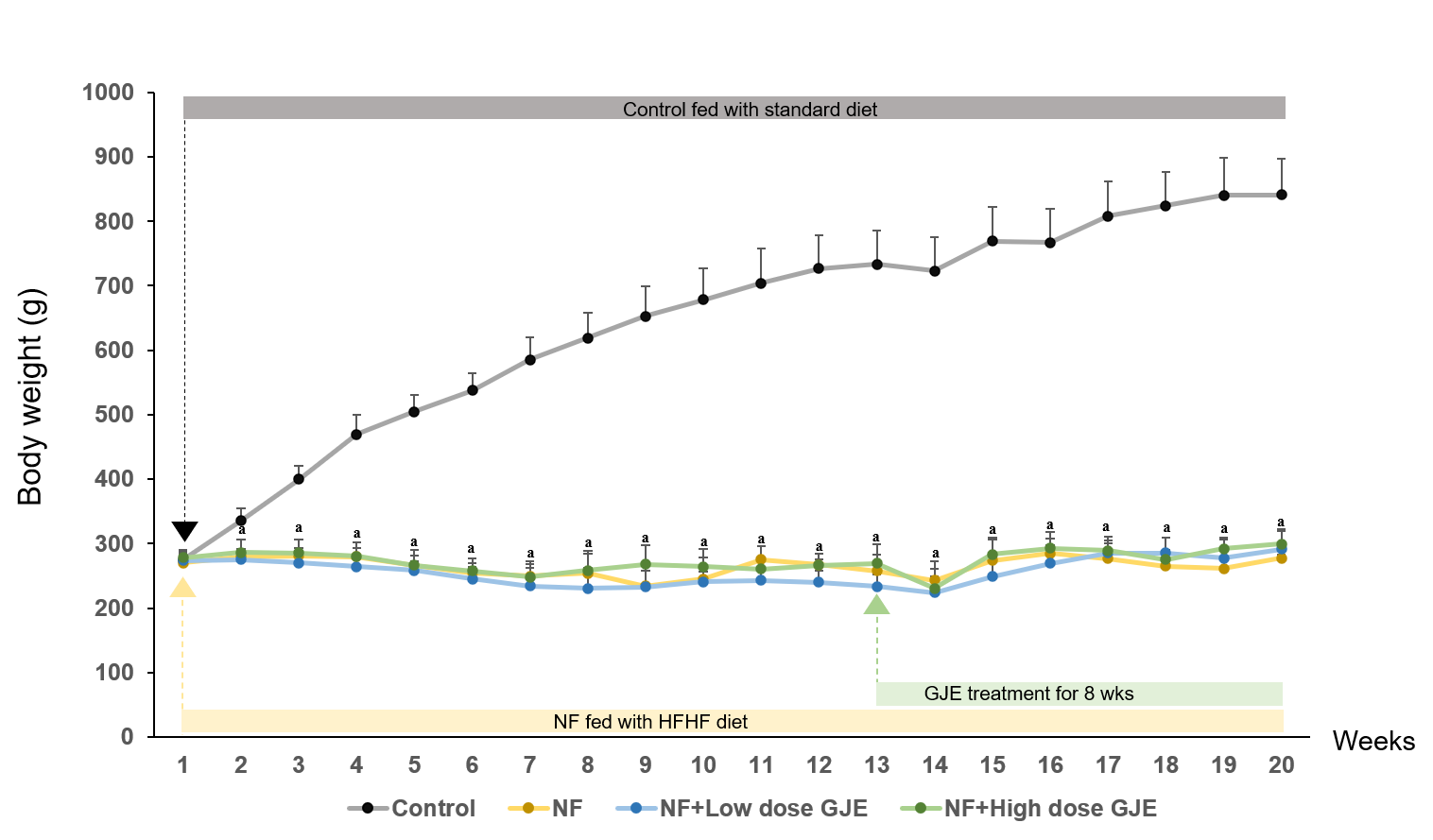

Supplement: S2 Fig — Control rats were fed a standard diet, whereas the NASH with fibrosis (NF) model was induced by a high-fat high-fructose (HFHF) diet. The graph shows weekly body weights for all groups over the 20-week period. Rats fed the HFHF diet exhibited lower body weights than controls from week 2 onward, with this difference was maintained throughout the study. However, no significant differences were observed among NF groups with or without GJE treatment. Data are expressed as mean ± SD (n = 6/group). a: p < 0.05 compared with the control group. (TIF) [file pone.0333800.s002.tif]

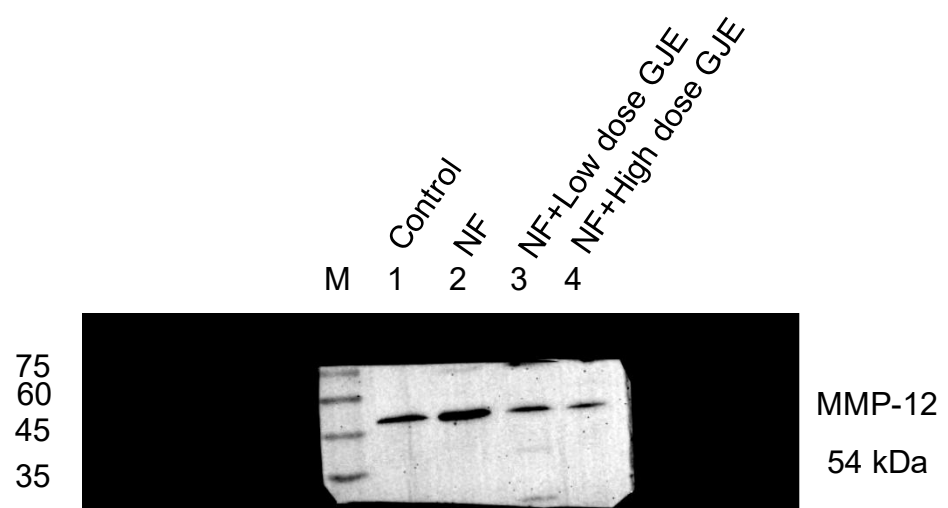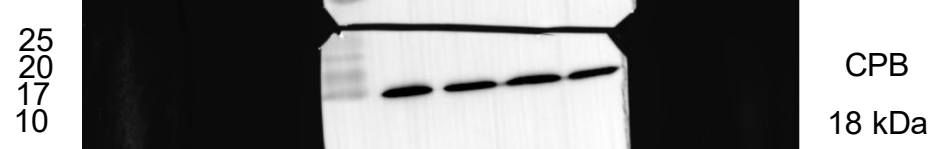

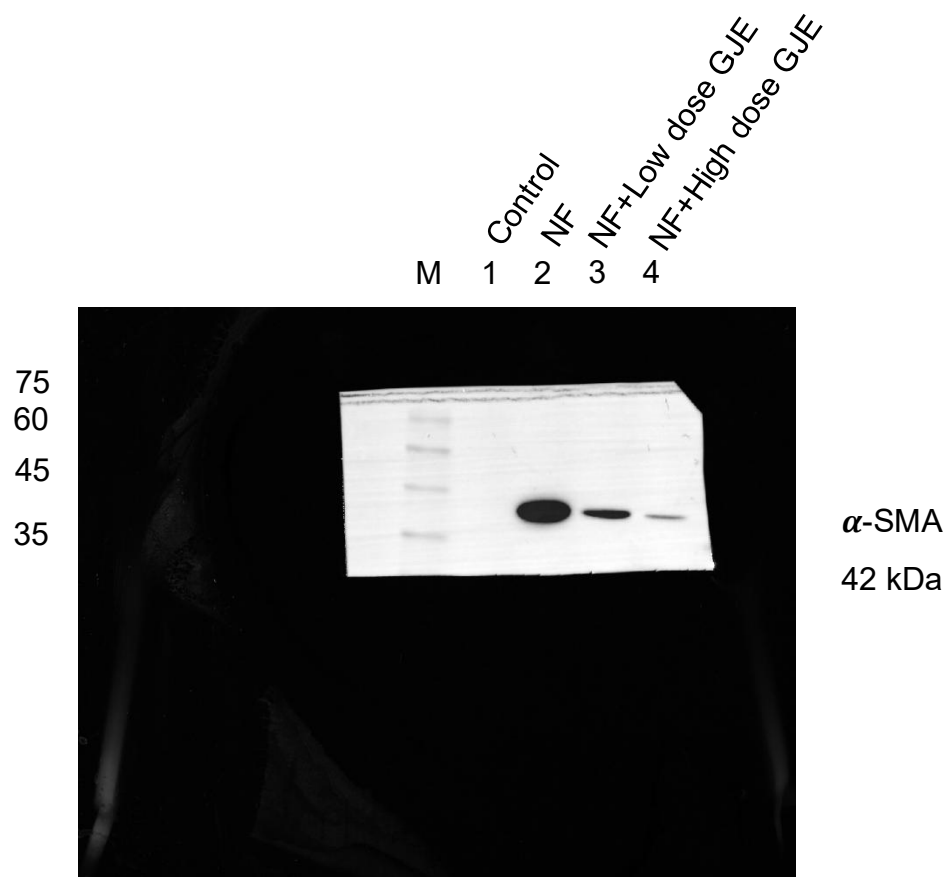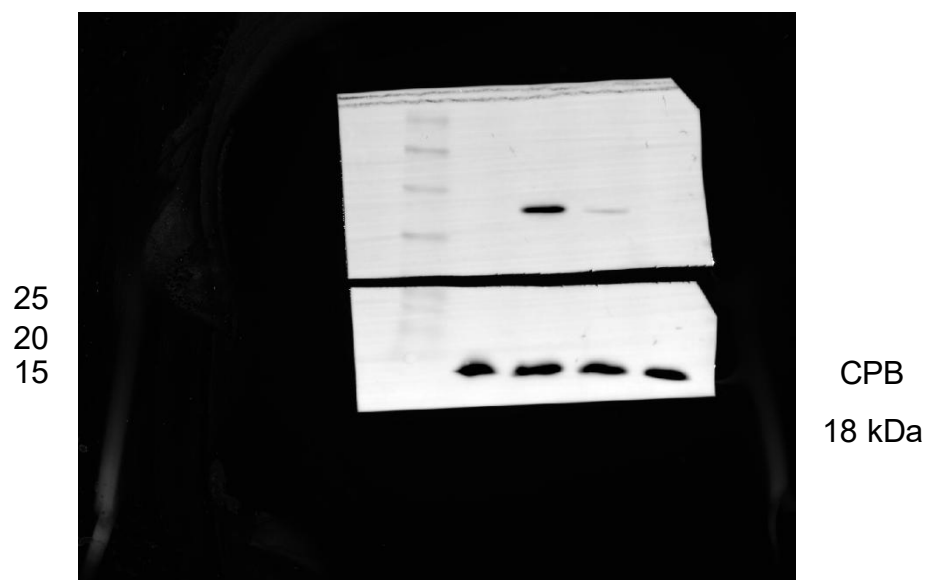

M      Control      NF      NF+Low dose GJE      NF+High dose GJE  
1      2      3      4

75  
60  
45  
35

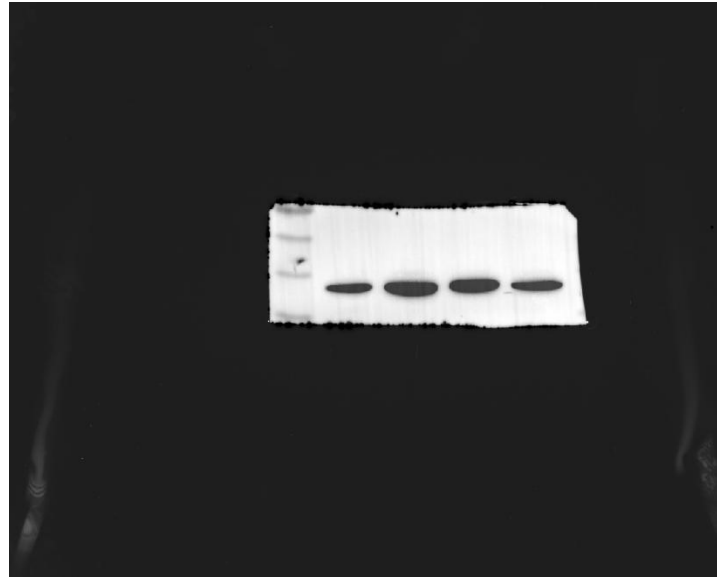

Beta actin  
43 kDa

25  
20  
15  
10

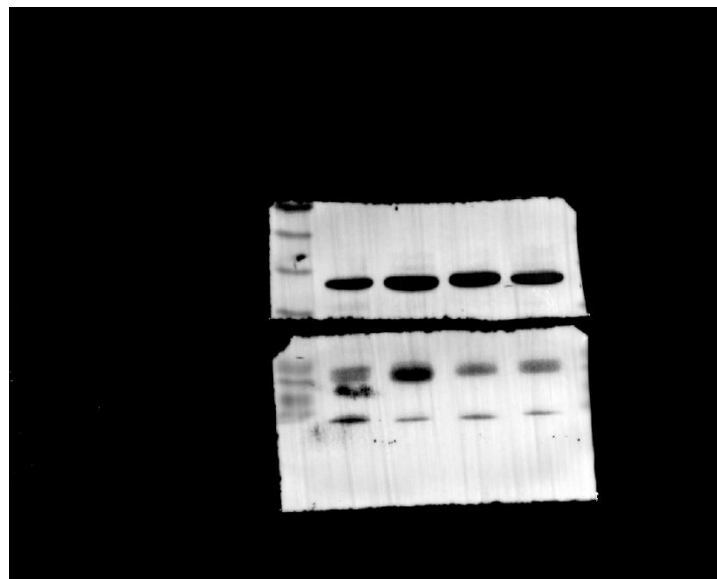

IL-13  
25 kDa

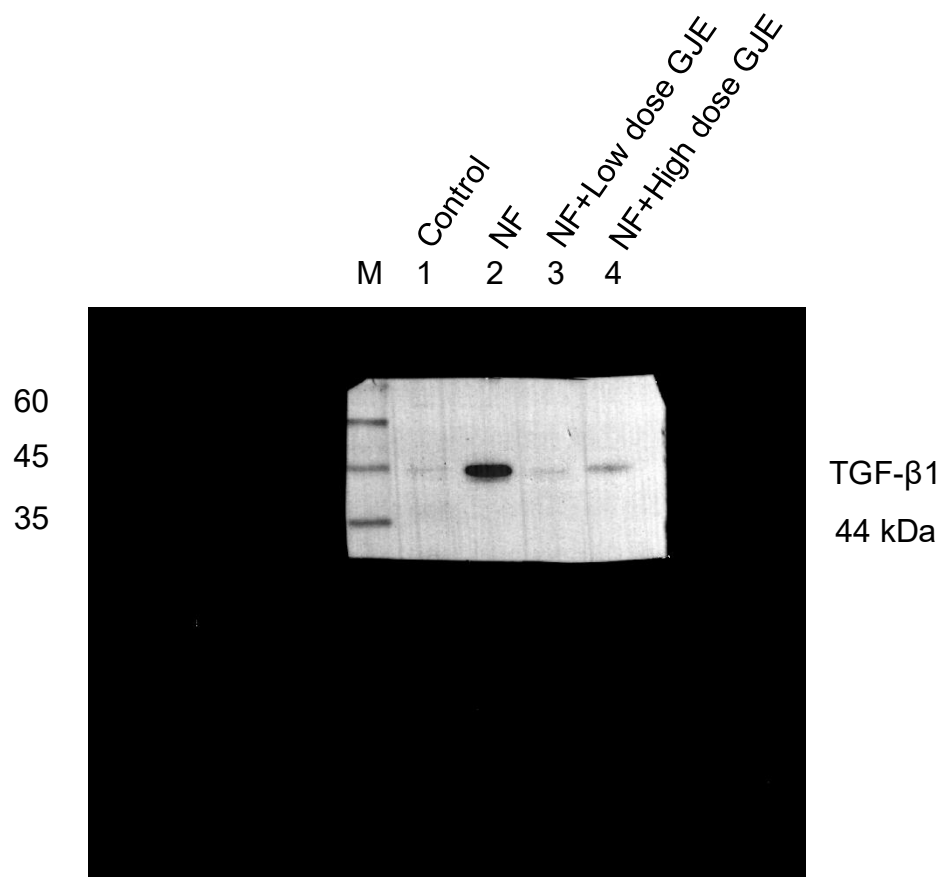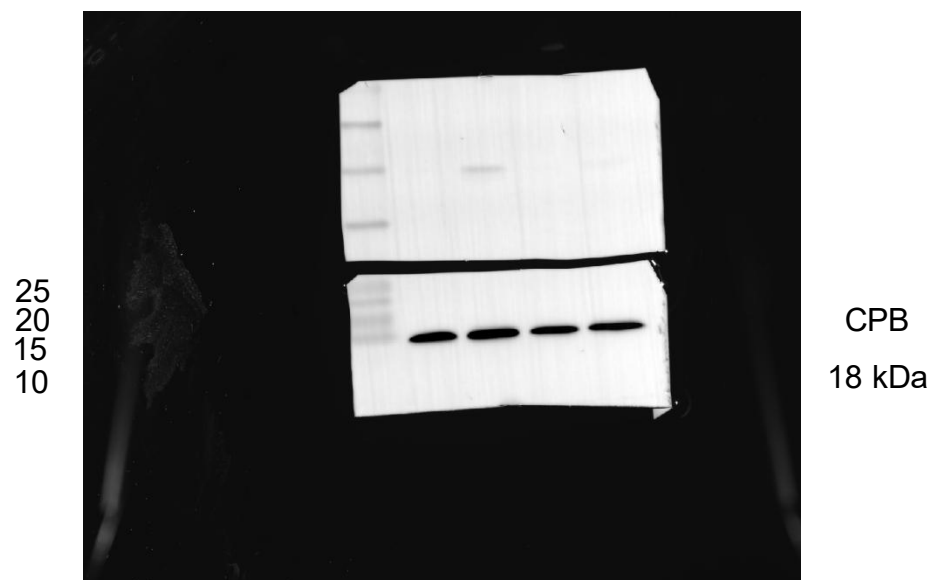

Supplement: S3 File — Lane 1 contains the PM2600 ExcelBand™ 3-Color High Range protein marker (SMOBIO, Taiwan), and lanes 2–5 correspond to Control, NF, NF + Low dose GJE, and NF+High dose GJE groups, respectively. Blots were developed using Clarity Western ECL substrates (BioRad, California, USA), captured with the Bio-Rad ChemiDoc Touch Imaging System, and quantified by Image Lab software (Bio-Rad, California, USA). Processed images were exported as TIF files for publication. (PDF) [file pone.0333800.s003.pdf]
